# Case report: Monitoring consciousness with fNIRS in a patient with prolonged reduced consciousness following hemorrhagic stroke undergoing adjunct taVNS therapy

**DOI:** 10.3389/fnins.2025.1519939

**Published:** 2025-02-04

**Authors:** Fei Gao, Likai Wang, Zhan Wang, Yaru Tian, Jingyi Wu, Mengchun Wang, Litong Wang

**Affiliations:** ^1^Department of Rehabilitation Medicine, The Second Hospital of Dalian Medical University, Dalian, China; ^2^Rehabilitation Center, Qilu Hospital of Shandong University, Jinan, China; ^3^University of Health and Rehabilitation Sciences, Qingdao, China

**Keywords:** disorders of consciousness, transcutaneous auricular vagal nerve stimulation, functional connectivity, functional near-infrared spectroscopy, stroke

## Abstract

Disorders of consciousness (DoC) resulting from severe brain injury present substantial challenges in rehabilitation due to disruptions in brain network connectivity, particularly within the frontal-parietal network critical for awareness. Transcutaneous auricular vagus nerve stimulation (taVNS) has emerged as a promising non-invasive intervention; however, the precise mechanisms through which it influences cortical function in DoC patients remain unclear. This study describes the effects of taVNS on fronto-parietal network connectivity and arousal in a 77-year-old female patient with unresponsive wakefulness syndrome (UWS). The patient received bilateral taVNS for 1 h daily over 3 months, with functional connectivity (FC) in the frontoparietal network assessed using functional near-infrared spectroscopy (fNIRS) and behavioral responsiveness evaluated through the Coma Recovery Scale-Revised (CRS-R). After taVNS intervention, mean FC was enhanced from 0.06 (SD = 0.31) to 0.33 (SD = 0.28) in the frontal-parietal network. The frontal-parietal were subdivided into 12 regions of interest (ROIs) and it was determined that the FC between the left dorsolateral prefrontal cortex (DLPFC) and the left prefrontal ROIs was 0.06 ± 0.41 before the intervention and 0.55 ± 0.24 after the intervention. Behavioral improvements were evidenced by an increase in CRS-R scores from 2 to 14, marking the patient's transition from UWS to minimally conscious state plus (MCS+). Additionally, regions associated with auditory and sensory processing showed increased cortical engagement, supporting the positive impact of taVNS on cortical responsiveness. This suggests its value as a non-invasive adjunctive therapy in the rehabilitation of DoC patients. Further studies are necessary to confirm these effects in a wider patient population and to refine the strategy for clinical application of taVNS.

## 1 Introduction

Severe brain injury can significantly disrupt the structural and functional connectivity (FC) within brain networks, leading to disorders of consciousness (DoC) (Giacino et al., 2014). DoC are marked by altered states of arousal and/or awareness. Patients with severely diminished arousal typically enter a coma—a state of profound brain function inhibition during the acute phase of injury. While coma often resolves as patients regain wakefulness within 2 to 4 weeks (Giacino et al., 2014), some patients (e.g., those with traumatic brain injury or stroke) do not progress to wakefulness due to extensive damage to the forebrain, thalamus, and brainstem, resulting in a prolonged DoC (pDoC) (Kondziella et al., 2020). Patients with pDoC are clinically classified based on the manifestations of conscious awareness into two primary categories: vegetative state/unresponsive wakefulness syndrome (VS/UWS) and minimally conscious state (MCS). Patients in MCS exhibit some level of conscious behavior (Naccache, 2018), whereas those in VS/UWS show no behavioral responses to external stimuli (Monti et al., 2010). MCS can further be divided into MCS minus (MCS-) and MCS plus (MCS+) (Bruno et al., 2011), depending on the presence of language-related behaviors (Thibaut et al., 2020). MCS may act as a transitional state for VS/UWS patients, potentially allowing a gradual return to wakefulness. The study concluded that patients with VS/UWS typically exhibit a lack of response to treatment due to extensive neural pathway disconnections in the forebrain (Edlow et al., 2021). Consequently, the focus of subsequent research was shifted to patients with MCS (Dong et al., 2023; Hu et al., 2024). However, premature palliative care for patients with VS/UWS may lead to a shift in focus from treatment to care during recovery, ultimately leading to a poor prognosis.

With advancements in central intervention techniques, including transcranial magnetic stimulation (TMS) and transcranial direct current stimulation (tDCS), new therapeutic hope has emerged for pDoC patients (Shou et al., 2021). Nevertheless, TMS is contraindicated in patients with intracranial metallic foreign bodies, aneurysms, and integration into the routine treatment of acute and chronic phases is challenging. Furthermore, TMS and tDCS directly stimulate specific cortical areas, including the dorsolateral prefrontal cortex (DLPFC), precuneus, and other brain regions associated with consciousness (Shou et al., 2021; Peng et al., 2023), as opposed to the broader activation of the forebrain. Invasive VNS (iVNS) has been demonstrated to promote the release of intracranial neurotransmitters, to have a widespread effect on the forebrain, to improve frontal-parietal FC, and to facilitate the restoration of consciousness (Thibaut et al., 2019). These findings offer a promising new avenue for treating patients with DoC. Nevertheless, implantation can result in complications such as peri-incisional haematoma and dyspnoea. Furthermore, the high cost of the necessary equipment and surgical procedures represents a significant barrier to its adoption in clinical practice. The advent of a novel stimulation paradigm, non-invasive VNS (nVNS), has rendered its utilization in a clinical setting more feasible, particularly in terms of portability and the incidence of adverse effects.

Neuroimaging evidence indicates that nVNS may have similar effects to iVNS (Badran et al., 2018). The nVNS is contingent upon the distribution of vagal afferent nerve fibers in the skin and is categorized into two distinct stimulation modalities: transcutaneous auricular VNS (taVNS) and transcutaneous cervical VNS (tcVNS) (Silberstein et al., 2016; Wang et al., 2023). TcVNS is predominantly left-sided stimulation, and this left-sided bias is largely due to the early FDA approval of left-sided VNS for the treatment of epilepsy, as well as the belief that stimulation of the right side of the tcVNS induces direct electrical input to the sinus node, which may increase the risk of adverse effects, such as arrhythmias (Morris and Mueller, 1999; Peng et al., 2023). In contrast, taVNS—whether applied on the left or right side—stimulates the auricular branch of the vagus nerve (ABVN), which sends signals to the nucleus tractus solitarius (NTS) via afferent fibers, thereby supporting integrative processing of information (Chen et al., 2015). The NTS subsequently transmits processed signals to the heart via bilateral cervical vagus nerves, thus avoiding direct stimulation of cardiac pacing points (Chen et al., 2015). Brain network configuration changes and altered FC in patients with DoC following severe brain injury may, however, complicate the bottom-up effects of VNS (Vitello et al., 2023). Additionally, complex neural reorganization post-injury may disrupt both afferent and efferent VNS pathways.

Bilateral taVNS has demonstrated initial efficacy in conditions such as major depression (Tu et al., 2018) and epilepsy (He et al., 2015), with minimal side effects and straightforward localization. Thus, in this study, we employed bilateral taVNS to promote arousal in patients with DoC, aiming to explore its potential mechanisms in facilitating consciousness recovery.

## 2 Enhancing consciousness in DoC: taVNS and the lateral frontoparietal network

Recent studies have demonstrated that the neuroprotective effects of taVNS can be achieved through anti-inflammatory mechanisms (Jiang et al., 2014; Kaczmarczyk et al., 2017), regulation of cerebral blood flow, reduction of blood-brain barrier permeability (Lopez et al., 2012; Yang et al., 2018), and modulation of the release of neurotrophic factors and neurotransmitters (Dong and Feng, 2018). In the treatment of DoC, Briand proposed the Vagal Cortical Pathways model, suggesting that taVNS activates the locus coeruleus and the raphe nuclei to release neurotransmitters such as norepinephrine and pentraxin, and extensively activates the lateral frontoparietal network (Briand et al., 2020). A study by Cavinato et al. (2015) demonstrated that simple sensory stimulation could enhance FC within the lateral frontoparietal network in patients with MCS, a response not observed in patients with VS/UWS. Interestingly, in this case, we observed that with the addition of taVNS treatment to conventional therapy, the patient showed enhanced FC of the frontoparietal network and showed clinical improvement. As Edlow noted in their review, the external frontoparietal network plays a pivotal role in consciousness (Edlow et al., 2021), which consists of the DLPFC and the posterior parietal cortices (PPC) (Zhou et al., 2016). This network is predominantly associated with external awareness, encompassing somatic sensory, visual and auditory perception. Given its foundational role in external awareness, the lateral frontoparietal network appears to be a crucial target in the rehabilitation of patients with DoC, potentially facilitating recovery of consciousness through its engagement with taVNS (Briand et al., 2020).

## 3 Long-term taVNS therapy enhances consciousness recovery in DoC patients

Currently, taVNS has been applied in the clinical management of DoC; however, its efficacy remains uncertain due to a limited understanding of the underlying neural mechanisms and substantial variability in patient responses. We reviewed nine existing trials that explored taVNS treatment for DoC patients (Yu et al., 2017, 2021; Hakon et al., 2020; Noé et al., 2020; Osińska et al., 2022; Yifei et al., 2022; Chen et al., 2023; Zhou et al., 2023; Wang et al., 2024). Our analysis revealed that patients receiving prolonged taVNS therapy demonstrated more substantial improvements in Coma Recovery Scale-Revised (CRS-R) scores and showed better prognoses (see Table 1). For example, in a case study by Osińska and colleagues, patients diagnosed with UWS for 2 years were treated with taVNS for 6 months. As a result, CRS-R scores improved from an initial 4–6 to 13, and the prognosis was favorable based on electroencephalography (EEG) results (Osińska et al., 2022). In contrast, a brief course of taVNS treatment, as demonstrated by Yifei, who treated 12 patients with DOC using taVNS for 4 weeks, revealed no improvement in CRS-R scores. However, the patients' EEGs exhibited an enhancement in their state of consciousness (Yifei et al., 2022). Accordingly, the authors posit that in practice, treatment-induced behavioral changes may be cumulative effects over time that are not immediately observable (Yifei et al., 2022). Other studies have demonstrated that a four-week course of taVNS is an effective method of accelerating the recovery of consciousness in patients with DoC, with a more pronounced effect observed in patients with MCS and a minimal effect in those with UWS (Yu et al., 2017, 2021; Noé et al., 2020; Zhou et al., 2023). Most of the current studies have treated patients with 4 weeks of taVNS (Yu et al., 2017, 2021; Noé et al., 2020; Yifei et al., 2022; Zhou et al., 2023; Wang et al., 2024), with fewer studies of long-term wake-promoting therapy. In response, we administered taVNS over 3 months to patients with a 50-day history of impaired consciousness. Following this intervention, patients transitioned from VS/UWS on admission to MCS+, with CRS-R scores improving from 2 to 14.

**Table 1:** Clinical study of taVNS in patients with DoC.

**References**	**Brain pathology**	**Stimulation side and site**	**Stimulation parameters**	**Stimulation protocol**	**Assessment**	**Security assessment**	**Clinical results**	**Side effects**
Yu et al. (2017)	Anoxia:1	Bilateral cymbal concha	20 Hz, < 1,000 us, 4–6 mA	4 weeks, twice a day, 30 min/day	CRS-R, 3.0 T fMRI	None	CRS-R: 6 (VS) → 13 (MCS), taVNS promotes enhanced FC of DMN	None reported
Noé et al. (2020)	TBI:7 Anoxia:4 Hemorrhage:3	Left tragus	Sinusoidal waveform, 20 Hz, 250 us, 1.5 mA	4 weeks, twice a day (5 days per week), 30 min/day	CRS-R	ECG	5 of 8 MCS patients: CRS-R↑	None reported
Hakon et al. (2020)	TBI: VS/UWS:3; MCS:2	Left cymbal conchae	25 Hz, 250 us, 30 s on/30 s off, with up to 0.5 mA for the first 3 days, and subsequently 1 mA for the remaining 8-week period	8 weeks	CRS-R	Portable patient monitor (IntelliVue MP5, Philips), pain assessment scores	1MCS → EMCS 1VS/UWS → EMCS 1VS/UWS → MCS	One patient experienced intermittent itching of the ear during stimulation
Yu et al. (2021)	Anoxia:5 Hemorrhage: 3 TBI: 2	Cymba conchae	20 Hz, 500 ms, 4–6 mA	4 weeks, twice a day (8:00 and 16:00), 30 min/day	CRSR, 3.0T ASL-FMRI	None	Boosted CBF in RtAS, not in nRtAS	None reported
Osińska et al. (2022)	TBI:1	Cymba conchae	25 Hz, 250 us, single-phase square wave, 30 s-30 s, 0.2 mA−1.5 mA	6 months, every day, 4 h/day	CRS-R, EEG	ECG	CRS-R: 4–6 → 8–13	ECG measures revealed a steady decrease in pre-stimulation HR combined with an increase in HRV-HR
Yifei et al. (2022)	taVNS group: 6 tnVNS group: 6	Bilateral auricular concha	20 Hz, < 1,000 us, 4–6 mA	4 weeks; twice a day 30 min/day	CRS-R, EEG	None	CRS-R: no change, In the taVNS group of MCS patients, delta and beta band energies were significantly altered in several brain regions, and cross-brain connectivity activity was also significantly altered	None reported
Vitello et al. (2023)	taVNS group: 22 sham group: 22	Bilateral cymba conchae	25 Hz, 200–300 μs, 3 mA, 30 s on/30 s off	5 day, 45 min/day	CRS-R, hd-EEG	NCS-R	None reported	None reported
Zhou et al. (2023)	tVNS group: 28, sham tVNS group: 29	Left outer ear	sinusoidal waveform, 20 Hz, 200 us	4 weeks, 30 min/session	CRS-R	Heart rate, breathing, pulse and blood pressure	Effective for MCS, not VS/UWS	Common side effects, unrelated to taVNS
Wang et al. (2024)	382 pDoC	Bilateral cymbal conchae	Sine wave, 200 μs pulse width, 20 Hz, 2 mA initial current intensity, 30 s on/30 s off	4 weeks, 6 days a week, twice a day, 30 min/session	CRS-R, FOUR, GCS, GOS-E	NCS-R, HRV, blood pressure	None reported	None reported

FOUR, full outline of unresponsiveness; GCS, Glasgow coma scale; GOS-E, extended Glasgow outcome scale; NCS-R, nociception coma scale-revised; HRV, heart rate variability.

## 4 Materials and methods

### 4.1 Ethics and patient

This study was conducted in the Department of Rehabilitation Medicine at the Second Hospital of Dalian Medical University, with ethical approval obtained from the hospital's Ethics Committee (approval number: KY2024-314-01). Informed consent for the study protocol was granted by the patient's legal guardian, who signed the consent form.

The patient was a 77-year-old woman with a hemorrhagic stroke and a CT scan of her head showed cerebral hemorrhages in her left parietal, occipital and temporal lobes. Injury was seen in the Splenium of the Corpus Callosum. Multiple foci of infarction were seen bilaterally in the basal ganglia region, pontine bridges, thalamic and radiocoronal areas. Fifty days after the hemorrhage, the patient was started on taVNS.

The patient met the following criteria: (1) diagnosis of VS/UWS; (2) CRS-R score of less than 3; (3) no improvement in CRS-R score after 30 days of pharmacological and central interventions; (4) normal blood pressure and electrocardiogram; (5) intact skin on the ear; and (6) no prior vagus nerve surgery.

### 4.2 taVNS stimulation

With the consent of the patient's family, we administered bilateral taVNS to the patient's cymbal conchae for a duration of 3 months. The treatment was conducted 5 days per week, with 1-h sessions held at the same time each day. A low-frequency pulsed electrical stimulator (En-stim4, Shanghai Xibei Electronic Science and Technology Development Co., Ltd.) was used in biphasic pulsed current mode for the taVNS application. The device was equipped with a sensor that triggered an alarm or stopped stimulation if there was insufficient electrode contact with the skin. The stimulation parameters included a frequency of 25 Hz, a pulse width of 300 μs, and an intensity of approximately 4–6 mA. Each stimulation phase lasted 30 s, followed by a 30-s rest period. The Nociception Coma Scale-Revised NCS-R (Chatelle et al., 2012) scale was used periodically to assess stimulation intensity, while real-time cardiac monitoring was conducted to ensure patient safety. In the event that the NCS-R score during stimulation exceeds 4, the current intensity of the taVNS will undergo a reduction of 0.2 mA, and the NCS-R test will be repeated. During stimulation, the heart rate increases or decreases to 5% or more of the basal heart rate. In this case, the treatment parameter will be adjusted downward by 0.2 until the heart rate returns to the baseline level.

### 4.3 Data acquisition and preprocessing

#### 4.3.1 Behavioral assessment

Consciousness was assessed by the patient's attending physician and rehabilitation rater using the JFK CRS-R. The scale comprises six subscales, namely auditory, visual, motor, verbal communication and arousal. The maximum score is 23, and scores below 8 are typically indicative of VS/UWS. To reduce the risk of misdiagnosis due to fluctuating levels of consciousness, five assessments were conducted over a period of 7 days following the patient's admission. Each assessment lasted a minimum of 20 min (Wannez et al., 2017). Given the patient's inability to sit independently, we conducted the assessment at the bedside, adjusting the bed to its maximum elevation to allow the patient to assume a seated posture.

#### 4.3.2 fNIRS assessment

##### 4.3.2.1 Resting-state data acquisition

**Figure 1:**
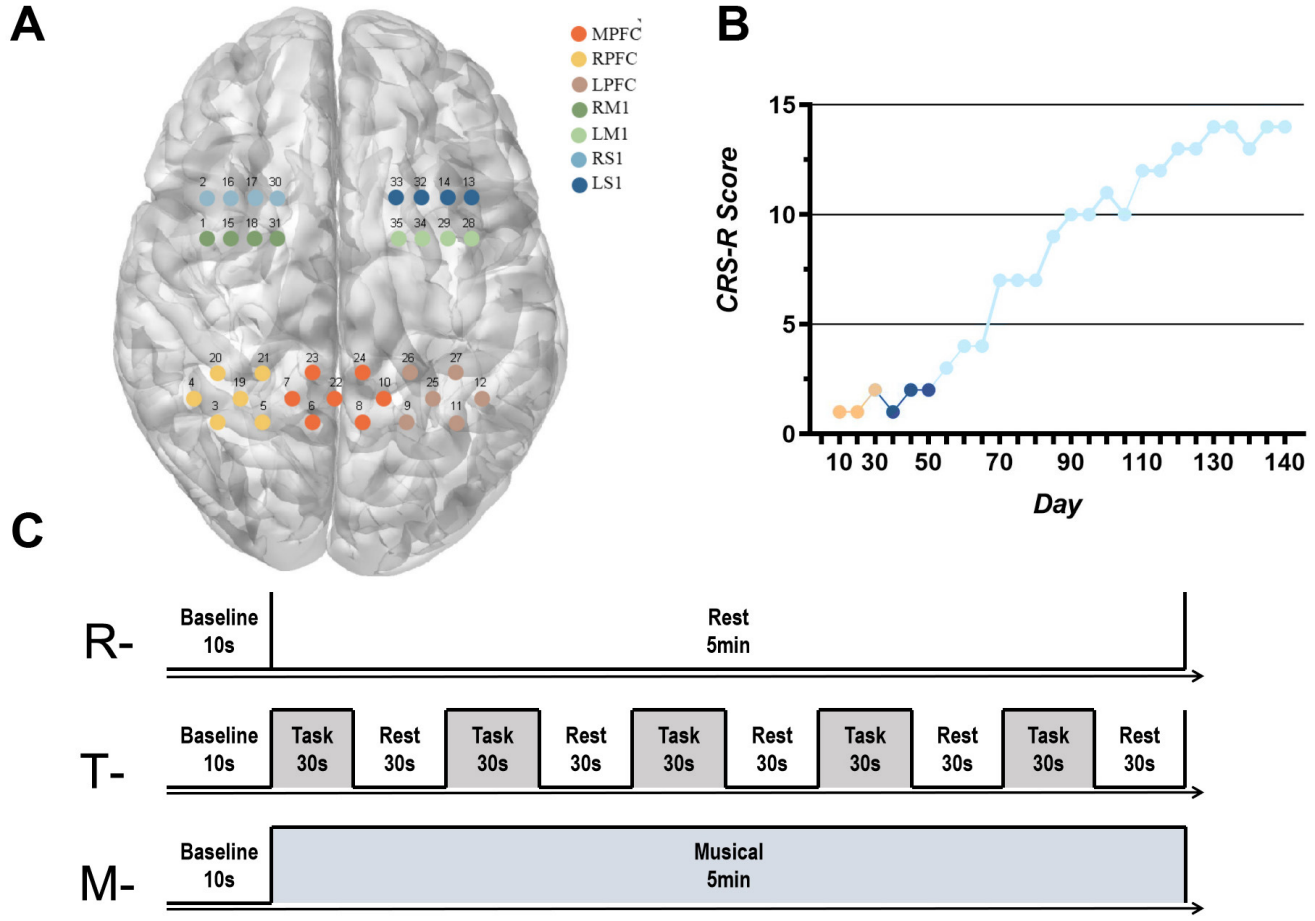
**(A)** Correspondence between fNIRS acquisition channels and brain networks; **(B)** CRS-R score: Orange indicates the patient's score within 30 days of injury, dark blue indicates the patient's score during routine clinical rehabilitation, and light blue indicates the patient's score during the addition of taVNS therapy. **(C)** fNIRS data acquisition test paradigm. “R”- Resting-state data acquisition, “T”-Task-state data acquisition, “M”- Musical stimulation for 5 min data collection.

We used a wireless continuous wave fNIRS system (NirSmart, Danyang Huichuang Medical Devices Co., Ltd., China) to detect residual consciousness in the patient's cortex. The acquisition head cap was designed based on the 10/20 international standard lead system, consisting of 24 probes and 24 light sources in 35 channels, covering the left and right frontal lobes (LFL/RFL), and the left and right parietal lobes (LPL/RPL). The distance between the sources and detectors is 30 mm. The position of the sources is shown in Figure 1A.

According to the MNI coordinates, the channels were divided into seven brain networks in the subjects' cerebral cortex: left prefrontal cortex (LPFC), middle prefrontal cortex (MPFC), right prefrontal cortex (RPFC), left primary motor cortex (LM1), right primary motor cortex (RM1), left primary sensory cortex (LS1) and right primary sensory cortex (RS1). The correspondence between the fNIRS acquisition channels and the brain network is shown in Figure 1A. The fNIRS has a sampling frequency of 11 Hz and two wavelengths, 730 and 850 nm, and can detect changes in the concentration of HbO (oxygenated hemoglobin) and HbR (deoxygenated hemoglobin) in the subject's brain in real time. Neuronal activity in the cerebral cortex leads to an increase in blood flow to the region, and there is evidence of a linear relationship between hemodynamics and neuronal activity (Wilcox and Biondi, 2015). Therefore, changes in neuronal activity can be assessed by measuring local concentrations of HbO, HbR, or HbT. The fNIRS recordings were performed by a trained therapist at the patient's bedside. fNIRS headcaps were placed on the patient, the patient was placed in a comfortable position raters turned off the room lighting and remained quiet before starting data collection. The entire measurement cycle comprises a 10-s baseline period, a 5-min data collection phase, and a final 10-s baseline period. The experimental paradigm is illustrated in Figure 1C.

##### 4.3.2.2 Task-state data acquisition

The auditory pathway is a relatively well-preserved information input channel in patients with DoC. Wojtecki et al. concluded that auditory stimulation typically activates the cerebral cortex in patients with DoC (Wojtecki et al., 2014). Therefore, the present study provided patients with auditory stimuli to detect the activation state of the cerebral cortex. The auditory stimuli consisted of several instructions and the experimental paradigm is shown in Figure 1C. The instructions are provided by the doctor in the form of verbal commands, requesting the patient to open and close his eyes. The instructions are delivered by the doctor in a continuous, standardized manner, with a 1-s interval between each instruction. Concurrently, 5 min of fNIRS signals were recorded, comprising 30 s of auditory stimulation and 30 s of rest. Each complete measurement cycle comprised a 10-s baseline measurement and five blocks. To ascertain whether the patient was following the instructions, another physician checked the patient's response to the auditory stimuli.

##### 4.3.2.3 Musical stimulation for 5 min data collection

The experimental paradigm, as illustrated in Figure 1C, comprises a baseline period of 10 s and a subsequent 5-min data collection phase. In order to minimize fatigue and to control for the total duration of the experiment, subjects were permitted to rest for a period of 10 min following the collection of task-state data for the stimulation of patient-preferred music (classical music) for a period of 5 min.

##### 4.3.2.4 fNIRS data processing

The preprocessing of the resting-state fNIRS (rs-fNIRS) data was conducted using the Preprocess module of the NirSpark software. Initially, motion artifacts were identified and removed through the utilization of spline interpolation, with a signal standard deviation threshold of 6 and a peak threshold of 0.5. Subsequently, general noise, including heartbeat, respiration, and Mayer waves, was filtered using a band-pass filter between 0.01 and 0.2 Hz. Subsequently, the path difference factor was set between −6 and 6, and the alterations in the concentration of HbO and HbR in the subjects' resting state were calculated in accordance with the modified Beer-Lambert law.

Resting-state data analysis: the Network module of the NirSpark software was employed to extract the changes in HbO and HbR concentrations over the time points measured in the resting state of the subjects. Furthermore, the Pearson's correlation coefficients of the HbO and HbR contents of the individual channels were analyzed over the time series. Subsequently, a Fisher Z transformation was conducted, with the transformed values defined as the strength of FC between channels.

We used Gretna (http://www.nitrc.org/projects/Gretna) (Wang et al., 2015) to set the sparsity threshold, thus binarizing the FC matrix to obtain the brain's functional network. Usually, the sparsity of a network is defined as the ratio of the number of edges available divided by the maximum possible number of edges in the network. We thresholded each correlation matrix repeatedly over a wide range of sparsity (0.1 ≤ S ≤ 0.45) at the interval of 0.01 (Luo et al., 2015). This range of sparsity was chosen to allow prominent small-world properties in brain networks to be observed. To compare the differences in brain network topological properties before and after treatment at different sparsities, we extracted five global property parameters using the functional brain network: small world properties (σ), clustering coefficient (Cp), shortest path length (Lp), global efficiency (Eg) and local efficiency (Eloc). Cp reflects the regional connectivity and grouping of the network; Lp is the average of the shortest path lengths between all pairs of nodes in the network; the shorter the shortest path lengths and the higher the global efficiency of the network, the faster the rate of transferring information between network nodes. In addition to the traditional small-world parameters (Cp and Lp), Eg and Eloc measure the ability of the network to transfer information at the global and local levels, respectively.

Task-state data analysis: the BlockAvg module in NirSpark is designed to perform block averaging analysis and linearity correction for tasks involving chunked designs. The means were calculated for the chunked tasks, which were repeated five times. This resulted in a 60-s block of tasks, with the first 30 s dedicated to the performance of the task and the subsequent 30 s allocated for rest. A general linear model (GLM) was employed to assess the relationship between alterations in blood oxygen levels and the temporal sequence of tasks. The haemodynamic response function (HRF) was employed as the basis for the design matrix. Baseline drift was eliminated and short-term correlations associated with cardiac and respiratory high-frequency noise were corrected. The developed design matrix was then fitted to the collected data. We performed a one-sample *t*-test on the patient's mean HBO concentrations in the 5 block tasks. The differences were accepted as significant when *p* < 0.05. Unless otherwise mentioned, the results are provided as (mean ± SD).

## 5 Results

### 5.1 Behavioral assessment

Before the start of treatment, the patient demonstrated dorsiflexion of the left calcaneus in response to strong painful stimuli and exhibited a blinking response to auditory cues, corresponding to a CRS-R score of 2. After the 3-month taVNS intervention, the patient was able to follow commands to shake and release her hand and showed a smiling response toward family members, resulting in an improved CRS-R score of 14 (Figure 1B).

#### 5.2 fNIRS assessment

##### 5.2.1 Average of frontal-parietal FC before and after treatment

**Figure 2:**
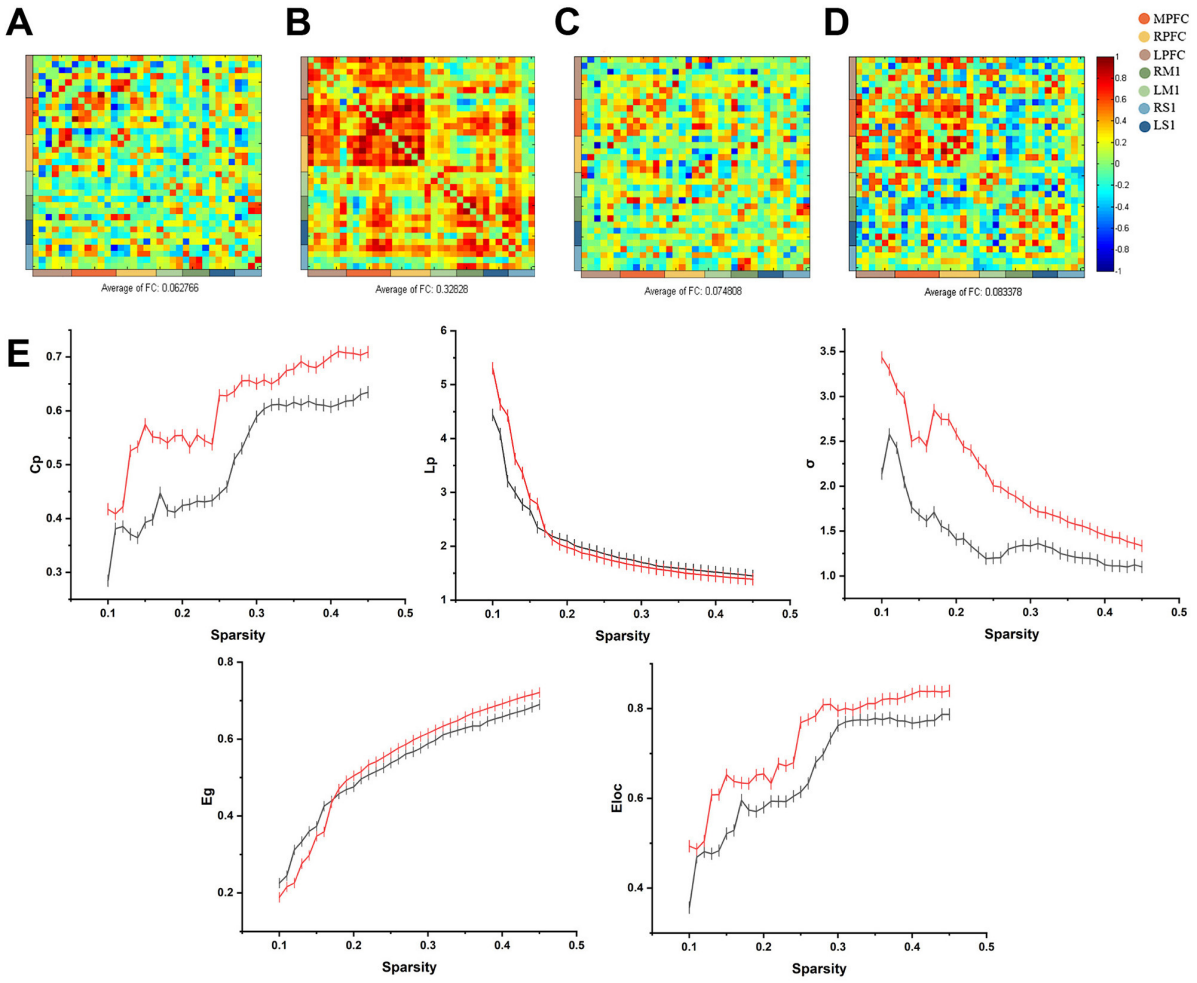
**(A)** Calculation of frontal-parietal FC before and after treatment based on HbO showed that the mean frontal-parietal FC before the intervention was 0.06 (SD = 0.31); **(B)** the mean of frontal-parietal FC after treatment was 0.33 (SD = 0.28); **(C)** Calculation of frontal-parietal FC before and after treatment based on HbR showed that the mean frontal-parietal FC before treatment was 0.07 (SD = 0.28); **(D)** the mean of frontal-parietal FC after treatment was 0.08 (SD = 0.34); **(E)** The relationship between curve of small-world correlation eigenvalues and sparsity. The red curve indicates the small-world correlation eigenvalues of patients before the taVNS treatment period, and the black curve indicates the small-world correlation eigenvalues after the taVNS treatment period.

Calculation of frontal-parietal FC before and after treatment based on HbO showed that the mean strength of frontal-parietal FC before treatment was 0.06 (SD = 0.31), and the mean strength of frontal-parietal FC after treatment was 0.33 (SD = 0.28) (Figures 2A, B).

Using HbR to calculate frontal-parietal FC before and after treatment, the average FC strength based on HbR before treatment was 0.07 (SD = 0.28), while after treatment, the average strength increased to 0.08 (SD = 0.34) (Figures 2C, D).

##### 5.2.2 Graph-theoretical topological analysis

The global network characteristics of the frontoparietal lobes are shown in Figure 2E. Cp, Eg and Eloc in both groups are overall positively correlated with sparsity positively correlated, and the LP and σ of the two groups decrease with increasing sparsity. Before treatment, Cp (0.5089 ± 0.0175), σ (1.4240 ± 0.0609), Lp (2.0912 ± 0.1647), Eg (0.5339 ± 0.0212) and Eloc (0.6604 ± 0.0204). In contrast, Cp (0.6010 ± 0.0145), σ (2.0766 ± 0.1006), Eg (0.5460 ± 0.0261), and Eloc (0.7301 ± 0.0181) were elevated, and Lp (2.0275 ± 0.1177) was decreased after treatment. The increase in Cp, decrease in LP and increase in σ between nodes of the brain network indicate that the communication efficiency of the brain network has been improved.

##### 5.2.3 Number of frontal-parietal connecting edges

FC with correlation coefficients greater than 0.6 were considered edges (Niu and He, 2014). At the level of HbO signaling, the number of connecting edges between the frontal-parietal was 30 before treatment and 115 after treatment. We observed an increase in the number of FC edges in the frontal-parietal lobe after treatment, from 9 to 46 for RPFC (CH 3, 4, 5, 19, 20) and from 0 to 17 for LS1 (CH 32, 33), whereas the number of edges in LPFC (CH 25, 26, 27) remained relatively stable.

##### 5.2.4 FC of resting-state ROI

**Figure 3:**
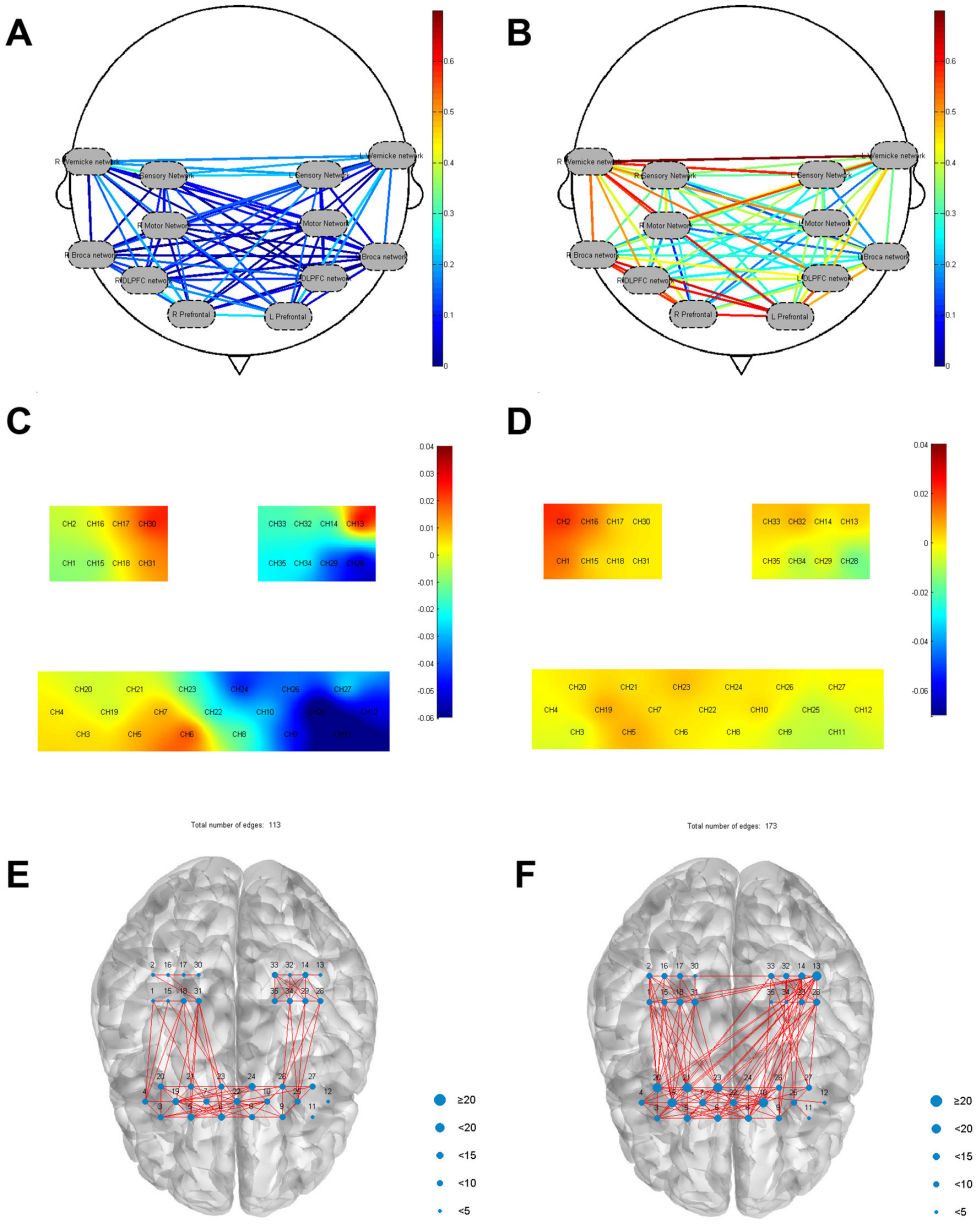
**(A)** FC between ROIs before treatment; **(B)** FC between ROIs after treatment; **(C)** Before treatment frontal-parietal task-state HBO mean value; **(D)** After treatment frontal-parietal task-state HBO mean value; **(E)** The number of frontal-parietal connecting edges stimulated by favorite music was 113; **(F)** The number of frontal-parietal connecting edges stimulated by disliked music was 173.

Regions of interest (ROI) based correlation analyses were conducted, with the brain's 35 channels divided into 12 ROIs following the framework of a previous study (Wang et al., 2023). Before treatment, FC between these sub-regions was generally low. However, post-treatment analysis revealed increased FC across various ROIs compared to the pre-treatment baseline. Before treatment, the FC between ROIs (mean ± SD) was 0.02 ± 0.39 for RM1 and right Wernicke, 0.02 ± 0.43 for right Wernicke and right Broca, 0.10 ± 0.27 for right Broca and right DLPFC, 0.14 ± 0.26 for right Broca and right prefrontal, and 0.06 ± 0.41 for left DLPFC and left prefrontal. After treatment, the FC between ROIs was 0.56 ± 0.21 for RM and right Wernicke, 0.52 ± 0.04 for right Wernicke and right Broca, 0.59 ± 0.27 for right Broca and right DLPFC, 0.57 ± 0.27 for right Broca and right prefrontal, and 0.55 ± 24 for left DLPFC and left prefrontal (Figures 3A, B).

##### 5.2.5 Mean value frontal-parietal HBO and HBR before and after task state treatment

The results showed differences in the distribution of patient's mean haemodynamic responses across multiple brain regions before and after treatment. Specifically, RPFC [(CH 5) (mean = 0.010, *p* < 0.05)], MPFC [(CH 6) (mean = 0.026, *p* < 0.05), (CH 7) (mean = 0.013, *p* < 0.05)], RM1 [(CH 18) (mean = 0.004, *p* < 0.05), (CH 31) (mean = 0. 014, *p* < 0.05)] activation was seen, and activation was also seen in the left Wernicke area [(CH 13) (mean = 0.042, *p* < 0.05)]. After treatment, MPFC [(CH 23) (mean = 0.013, *p* < 0.05), (CH 10) (mean = 0.007, *p* < 0.05)], RPFC [(CH 19) (mean = 0.010, *p* < 0.05)], RS1 [(CH 17) (mean = 0. 007, *p* < 0.05)], RM1 [(CH 1) (mean = 0.016, *p* < 0.05), (CH 15) (mean = 0.003, *p* < 0.05)], right Broca [(CH 19) (mean = 0.010, *p* < 0.05)], were observed to activate during the task. As shown in Figures 3C, D.

##### 5.2.6 Number of frontal-parietal connecting edges with musical stimulation for 5 min

The patient's preferred music was played for 5 min, followed by music that the patient disliked for another 5 min. The intervals between each play were 10 min to avoid the superimposed effect of the previous stimulus. It was found that a higher number of edges were found when playing the disliked music (edge count = 172) compared to the resting state 5 min test (edge count = 30), in particular an increase from 3 to 61 in Broca's area (CH 19, 20, 21) and from 0 to 24 in Wernicke's area (CH 13). Prefrontal and bilateral motor and sensory connections also increased more than resting state 5 min test. There was also an increase in the number of edges in patients when favorite music was played (edges = 113), but not as pronounced as when disliked music was played (Figures 3E, F).

## 6 Discussion

In this study, taVNS was administered for 1 h daily over 3 months to a patient diagnosed with VS/UWS. We observed that after approximately 90 days of stimulation, the patient's CRS-R score improved from an initial score of 2 to 14 and the diagnosis changed from VS/UWS to MCS+. Notably, taVNS was associated with minimal or no adverse effects on the patient. To investigate the potential modulatory effects of taVNS on neural activity in patients with DoC, we analyzed seven brain networks, examined changes in resting-state FC across 12 ROI, and compared mean HbO and HbR levels in each brain region during task states before and after 3 months of taVNS treatment. The results showed enhanced FC between the frontal and parietal cortices after treatment compared to before treatment. These findings suggest that taVNS may aid in consciousness recovery by enhancing FC between the frontal and parietal cortices.

The human brain is a complex, interconnected network system with a variety of important topological properties. Among them, the brain functional network is a typical small-world network (Li et al., 2019). The characteristics of high Cp and short Lp are conducive to the efficient transmission of information in the network (Suo et al., 2015). Functional segregation and integration are two major organizational principles of the human brain, the coexistence of which ensures the effective integration of multiple segregated sources of information in the brain (Sporns, 2011). Despite the fact that the functional brain networks demonstrated small-world properties both before and after treatment, Cp and Eloca exhibited smaller pre-treatment relative to post-treatment values across a wide range of sparsity levels. Lower pre-treatment Cp and Eloca values are indicative of patients exhibiting reduced functional segregation and less efficient and more unstable local messaging in the functional network (Luo et al., 2015). Eloc is thought to correlate with information processing and network fault tolerance between neighboring regions, and low Eloc usually corresponds to reduced fault tolerance and diminished immunity to interference in brain networks (Bullmore and Sporns, 2009). In this study, Cp, σ, Eg, and Eloc increased and Lp decreased as patient's level of consciousness improved after treatment, which is consistent with previous studies (Liu et al., 2023a).

The PFC has been identified as a pivotal center in higher cognitive functions in humans, including emotion regulation, attention and the formation of working memory (Farzan et al., 2009). In this study, we observed extensive activation in the PFC of patients after treatment by fNIRS. Several bedside assessments conducted before treatment showed no observable response to auditory stimuli, including family members calling or startle responses. However, when assessing consciousness using task-state fNIRS, we observed a sustained increase in mean HbO concentration in Wernicke's area (CH 13) during auditory tasks. Additional activations were noted in the RPFC (CH 5) and the RM1 (CH 18, 31). After treatment, activation in the RPFC (CH 19) became more extensive and intense, with significant activation also observed in the RS1 (CH 2, 16, 17), RM1(CH 1, 15), Broca's area (CH 19) and left Wernicke's area (CH 13). In addition, the present study found that patients could receive the physician's commands and activate the appropriate motor areas for feedback, possibly due to an impaired motor output pathway, which resulted in a diagnosis of unresponsiveness as the physician could not clinically observe the patient's motor output. Yifei showed that patients retaining hearing are more favorable for disease recovery (Yifei et al., 2022). In this study, the patient's response to instructions was detected using fNIRS and it can be surmised that the patient retained some auditory function and likewise the patient had a better prognosis.

pDoC are complex and refractory conditions, with an unclear pathogenesis that limits the optimization of therapeutic strategies. The precise mechanisms by which taVNS influences central nervous system (CNS) circuits are also not yet fully understood (Thibaut et al., 2019). This gap in understanding hinders the development of targeted therapeutic options. Notably, FC between the frontal and parietal brain regions has been shown to correlate significantly with the level of consciousness in DoC patients (Giacino et al., 2014). Studies further suggest that patients with VS/UWS can be distinguished from those in MCS based on interhemispheric connectivity within the frontoparietal cortex (Chennu et al., 2017). According to the mesocircuit hypothesis (Schiff, 2010), the frontal and parietal cortices act as critical hubs, supporting consciousness-related information processing within the cortical level of the consciousness circuit through frontoparietal connections. The frontal cortex plays a central role in orchestrating goal-directed behavior, regulating arousal across varying states, and activating the central thalamus through descending projections (Nee, 2021). This activation enables the brain to respond to higher cognitive demands. Additionally, parietal involvement has shown a significant correlation with levels of consciousness, indicating its role in supporting conscious states (Chennu et al., 2017). The FC between the frontal and parietal cortices enables these two cortices to regulate not only the mesocircuit directly through feedback but also indirectly through the frontal cortical-striatopallidal-thalamocortical loop systems (Schiff, 2010). This allows for the maintenance of normal conscious pathways in the brain (Liu et al., 2023b).

In clinical practice, the gold standard for assessing levels of consciousness in DoC patients is the CRS-R, which aids in differentiating between VS/UWS and MCS (Giacino et al., 2004). However, the CRS-R relies on the clinician's subjective interpretation of the patient's reflexive and non-reflexive behaviors in response to external stimuli. This subjectivity introduces variability, as different raters may assign different scores, and even the same rater might produce varying scores due to fluctuations in the patient's level of consciousness. Research indicates that up to 40% of patients with uncommunicative DoC may be misdiagnosed as being in VS/UWS, and inaccurate assessments of VS/UWS permanence can lead to a focus on management rather than rehabilitation, resulting in poorer prognoses (Wannez et al., 2017; Bodien et al., 2024). Furthermore, the evaluation of non-reflexive behaviors may not directly reflect the patient's true level of consciousness, as fluctuating consciousness levels, cognitive impairments (e.g., aphasia, dysarthria), sensory deficits (e.g., blindness, deafness), motor dysfunction, low activity levels, fatigue, and pain can all impact assessment accuracy (van Ommen et al., 2018). Consequently, DoC patients with motor or sensory impairments are at risk of misdiagnosis. Additionally, because CRS-R is based on behavioral assessment, it does not account for changes in brain function and thus cannot detect intrinsic improvements in consciousness. In a study, a large number of active consciousness paradigms in patients with DoC were investigated using EEG (Yifei et al., 2022). The study found that, despite an absence of visible signs of consciousness, 15% of VS/UWS patients were able to comply with instructions by modulating their brain activity. This highlights an urgent need for new methods to objectively and quantitatively assess consciousness from the perspective of brain function to enhance clinical diagnostic accuracy. fNIRS shows promise in providing insights into cognitive processes that may not be discernible through traditional behavioral assessments (Li et al., 2021; Lu et al., 2023). fNIRS is a non-invasive technique that monitors conscious brain activity by measuring near-infrared light absorption in cerebral tissue (Eastmond et al., 2022). Due to the differing absorption spectra of HbO and HbR at wavelengths between 650 and 950 nm, fNIRS can detect changes in their relative concentrations through diffuse light scattering, thereby inferring brain activity (Almajidy et al., 2020). Compared to EEG/MEG, fNIRS offers higher spatial resolution (1–2 cm), and compared to fMRI, it provides superior temporal resolution (milliseconds). Most importantly, fNIRS is portable, quiet, cost-effective, easy to administer, and tolerates movement artifacts and varied environments, making it ideal for prolonged or repetitive bedside measurements in DoC patients (Song et al., 2018). Therefore, fNIRS-related metrics offer a viable method for assessing the effects of taVNS on brain function in DoC patients.

When analyzing subdomains of the CRS-R scale, we observed that improvements in patient scores were primarily centered on visual and auditory function. Using an auditory paradigm, we examined cortical responses to auditory stimuli in patients with DoC to explore potential correlations between these responses and patient outcomes. Our study found that musical stimulation activates the frontal and parietal cortices, which subsequently enhances FC within the cerebral cortex, supporting the hypothesis proposed by Wojtecki that simple somatosensory, auditory, and visual stimuli typically activate primary cortical areas in VS/UWS patients (Wojtecki et al., 2014). Beyond speech, music is one of the most versatile and complex auditory stimuli, engaging auditory, visual, and somatosensory processing systems (Rollnik and Altenmüller, 2014). In this study, different types of music were played for patients, revealing variations in cortical connectivity edges based on the type of music. This suggests that the brain may process different music types in a basic manner that elicits arousal and emotional responses, with emotional improvement potentially linked to functional recovery, as observed in stroke patients. Several previous studies have reported that in healthy subjects, listening to music induces arousal and mood changes in the listener, which improves the listener's cognitive abilities (e.g., verbal processing, visual attention, etc.) (Soto et al., 2009; Gómez-Gallego et al., 2021), and there is neuroimaging evidence to support this notion, whereby listening to pleasing music activates limbic and paralimbic structures (e.g., the hippocampus), as well as structures involved in reward circuits (e.g., nucleus of the arcus accumbens), leading to a person's feeling of comfort and calm (Särkämö et al., 2008; Bleibel et al., 2023). Similarly, musical stimulation has been reported to induce broad cortical and subcortical activation in DoC patients, particularly in the bilateral frontal, temporal, parietal, and cerebellar regions, with strong responses in the frontal cortex, cingulate gyrus, amygdala, and hippocampus—areas closely associated with emotion (Rollnik and Altenmüller, 2014). In this study, musical stimulation in DoC patients enhanced bilateral FC between the frontal and parietal cortices, with the most pronounced effect seen when patients listened to their dislike music. It is hypothesized that music influences neural networks, enhances synaptic plasticity, and improves perceptual processing (Sihvonen et al., 2017). Therefore, music therapy-especially with dislike music-could potentially be employed to promote arousal during the early neurological rehabilitation of DoC patients. Further research is needed to establish the therapeutic potential of music therapy for early rehabilitation in patients with coma, VS/UWS, or MCS.

## 7 Conclusion

This study attempted to explore the evidence for the benefit of bilateral taVNS for VS/UWS treatment lasting 90 days. We observed a gradual increase in patient's CRS-R scores during taVNS therapy. The frontal-parietal FC of the patients was enhanced after treatment compared with before treatment, and we speculate that this may be one of the reasons for the improvement in the patient's consciousness. Therefore, we hypothesized that long-term taVNS treatment may be an adjunctive therapy for impaired consciousness due to hemorrhagic stroke. Further studies in larger patient populations are recommended to confirm these findings and refine the taVNS protocol in clinical practice.

## 8 Limitations and future directions

This study has several limitations. First, the small sample size limits our ability to conclusively attribute improvements in consciousness to taVNS alone, as we cannot fully rule out the possibility of spontaneous recovery. Additionally, fNIRS has its own limitations; for instance, it cannot assess subcortical brain function recovery or activation levels in the thalamus and brainstem. Future research should incorporate multimodal assessments of DoC patients and further explore the effects of taVNS on subcortical FC in this population. To establish the efficacy of taVNS, larger-scale randomized controlled trials are necessary. Such studies should aim to optimize the stimulation protocol, evaluate whether binaural taVNS is more effective than monaural stimulation, and determine whether therapeutic effects vary according to etiology and lesion type.

## Data Availability

The raw data supporting the conclusions of this article will be made available by the authors, without undue reservation.
